# Hidden Choroidal Melanoma Presented with Bullous Non-rhegmatogenous Retinal Detachment in a Myopic Asian Lady: A Rare Manifestation

**DOI:** 10.7759/cureus.4335

**Published:** 2019-03-27

**Authors:** Norihan Ibrahim, Zakariah Sakinah, Zulkifli Abdul Ghani, Mohtar Ibrahim

**Affiliations:** 1 Ophthalmology, School of Medical Sciences, Universiti Sains Malaysia, Kota Bharu, MYS; 2 Ophthalmology, Hospital Raja Perempuan Zainab II, Kota Bharu, MYS; 3 Ophthalmology, School of Medical Sciences, Universiti Sains Malaysia, Kubang Kerian, MYS

**Keywords:** rhegmatogenous retinal detachment, exudative retinal detachment, choroidal melanoma, non-caucasian

## Abstract

Choroidal melanoma is an uncommon malignant melanoma among non-Caucasians. We report here a case of a high myope patient who presented with symptoms of acute retinal detachment, which had been diagnosed as possible rhegmatogenous retinal detachment from the initial assessment. A detailed vitreoretinal evaluation revealed a glimpse of an obscured intraocular mass underneath the detached retina, which later proved to be a choroidal melanoma. This is an unexpected cause of retinal detachment in a myopic eye. Furthermore, the rare nature of choroidal melanoma in this particular region of the world makes this an ignored diagnosis at presentation.

## Introduction

Malignant melanoma of any type is uncommon in non-Caucasians. This includes uveal melanoma, the most common non-cutaneous malignant melanoma, with an incidence of 5%-7% of melanomas [1]. However, the incidence varies by sex, race, and country. Males were found to have a 30% greater incidence than females [2-3].

As a whole, melanoma is a relatively rare tumor, arising from melanocytes from various anatomic locations, including skin, mucous membrane, ocular regions (uvea, conjunctive, eyelid, and orbit), and, infrequently, from unknown primary sites [4].

In adults, a secondary malignant lesion contributes as the most common intraocular tumor. While among primary intraocular malignancy, a uveal melanoma was found to be the leading etiology, with a mean age-adjusted incidence of 5.1 cases per million per year. However, many other benign and malignant lesions can mimic its ophthalmoscopic features [5]. The lesion predominantly involves the choroid, followed by the ciliary body and iris. Other than being Caucasian, the host susceptibility factors include fair skin, light eye color, inability to tan, ocular or oculodermal melanocytosis, cutaneous, iris, or choroidal nevus, and BRCA1-associated protein-1 mutation [6].

Exudative retinal detachment not only has a strong association of choroidal melanoma, but it also has been reported as the major cause of visual loss in uveal melanoma [7]. It is a non-specific occurrence, as the etiology is wide such as following injury, inflammation, or vascular abnormalities. In those bearing this tumor, localized subretinal fluid is usually observed over the tumor or gravitating from the tumor to the dependent portion of the eye [8].

Contrary to exudative retinal detachment, the co-presentation of choroidal melanoma and rhegmatogenous retinal detachment is very rare, accounting for less than 1% of the total choroidal melanoma cases. More commonly, the choroidal mass simulating choroidal detachment leads to misdiagnosis and initiation of inappropriate treatment [9-10].

## Case presentation

A 55-year-old Malay lady with underlying myopia of both eyes sustained high myopia over the right eye with a -9.00 spherical dioptre and moderate myopia over the left eye with a -5.00 spherical dioptre. She presented with a two-week history of sudden-onset reduced vision over the left eye, associated with flashes of light and superior field defect. Otherwise, she denied any preceding trauma and no similar history over the fellow eye. She was relatively well with no constitutional symptoms. She was assessed at a district hospital and referred to the vitreoretinal center for rhegmatogenous retinal detachment after primary review noted that she had inferior retinal detachment with suspicion of a horseshoe tear present at the 6 o'clock position.

**Figure 1:**
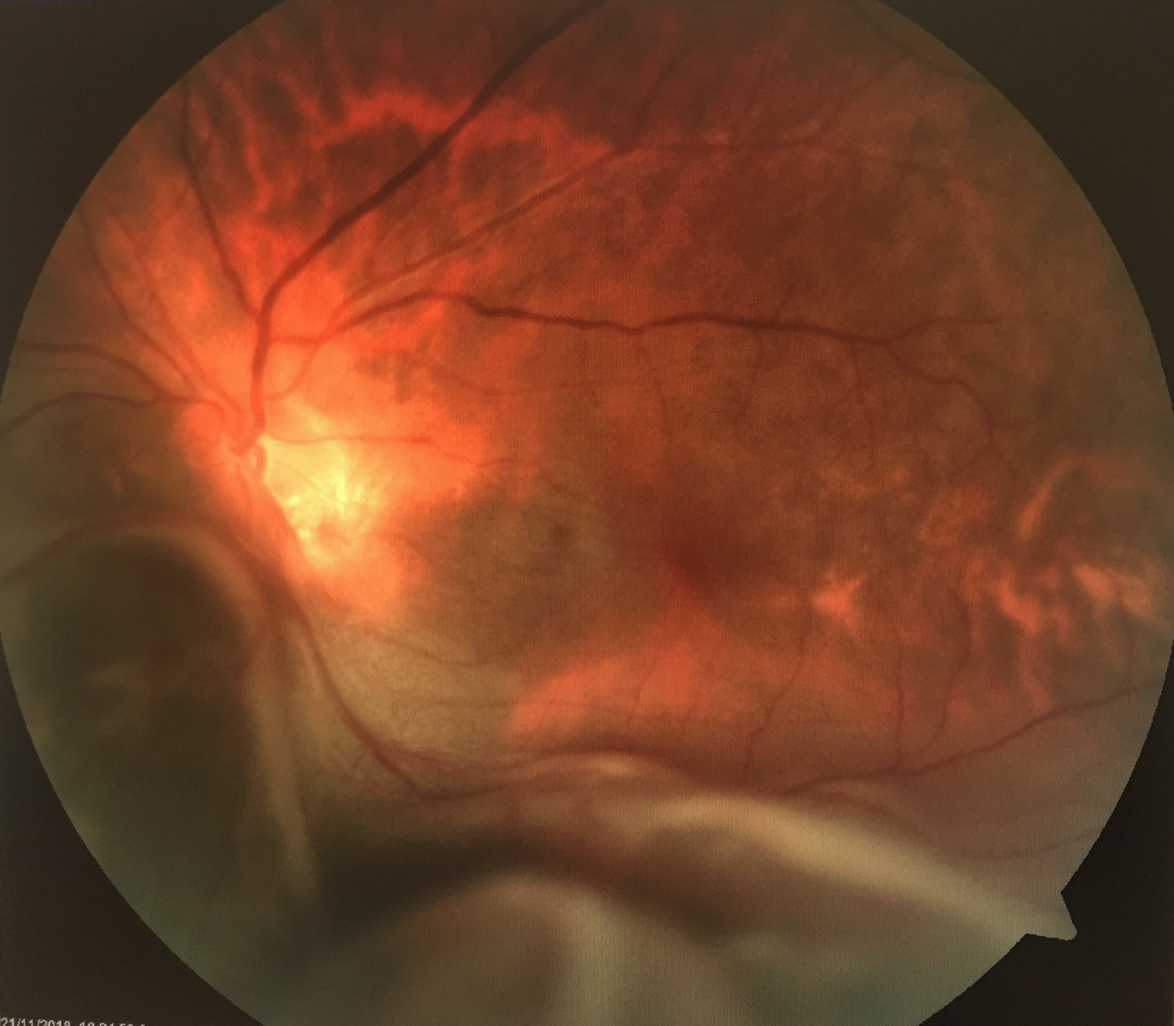
Fundus photography of the left eye showed huge inferonasal exudative retinal detachment.

**Figure 2:**
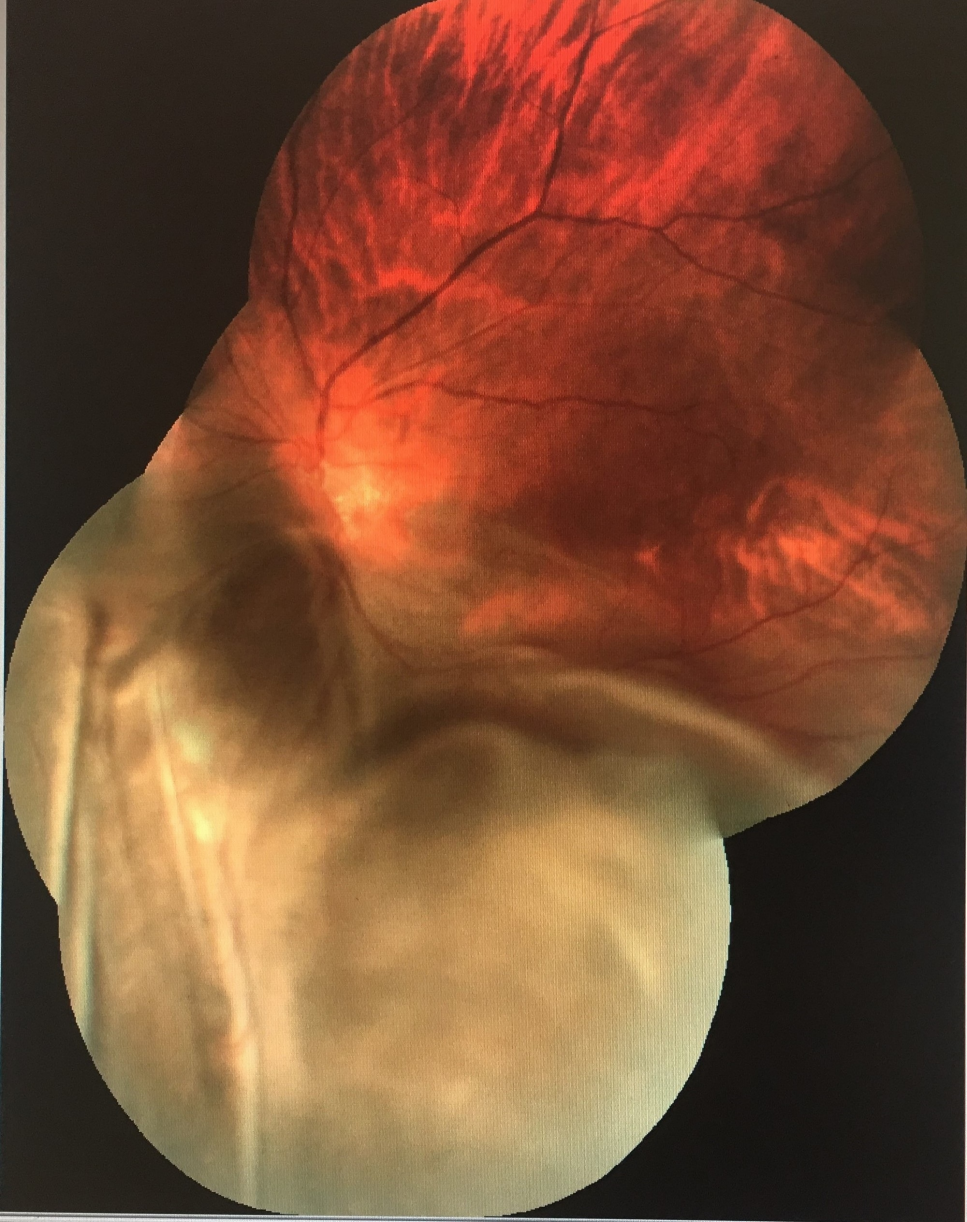
Wider view of left fundoscopy showing the extension of the exudative retinal detachment

On examination, the patient had moderately tanned skin. Her visual acuity was 6/18 with a pinhole of 6/12 over the right eye and 6/60 over the left eye. Relative afferent pupillary defect (RAPD) was absent. An anterior segment examination was unremarkable. Intraocular pressure was 16 mmHg over the right eye and 14 mmHg over the left eye. Posterior segment examination showed myopic fundus bilaterally, with a tilted optic disc of the right eye. Fundus examination of the left eye showed the presence of bullous retinal detachment, extending from 5 o'clock to 9 o'clock, with shallow detachment over the macula (Figures 1-2). The left optic disc was partially obscured by the detachment. A detailed examination with indentation by a vitreoretinal surgeon revealed a suspicious mass underneath the detached retina of the left eye, with no visible tear seen. The posterior segment of the right eye was otherwise normal. The systemic examination was also unremarkable with clear lungs, no palpable lymph nodes or breast lump, no palpable abdominal mass, and no hepatosplenomegaly.

**Figure 3:**
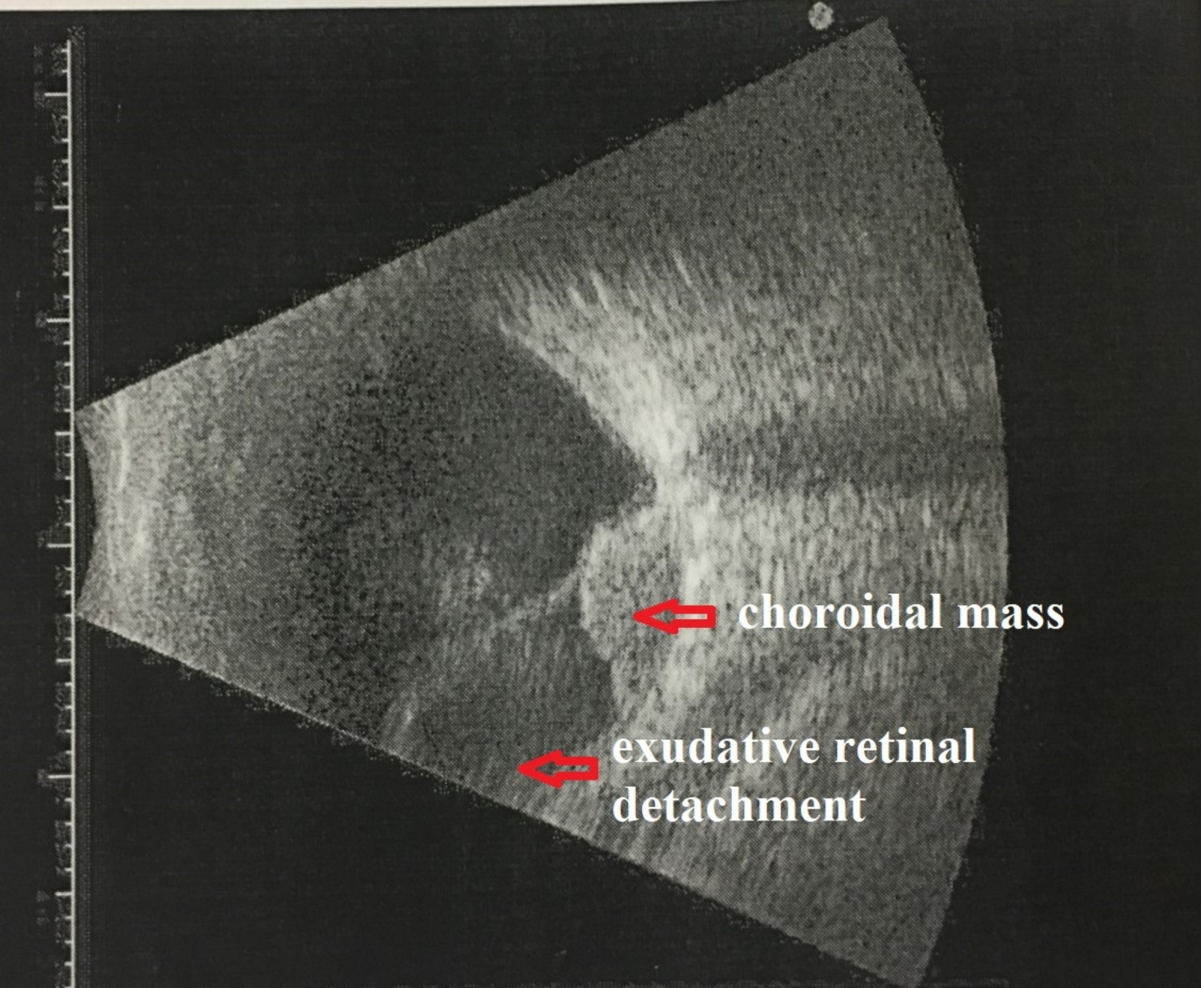
B-scan showed the presence of the choroidal mass underneath the detached retina

**Figure 4:**
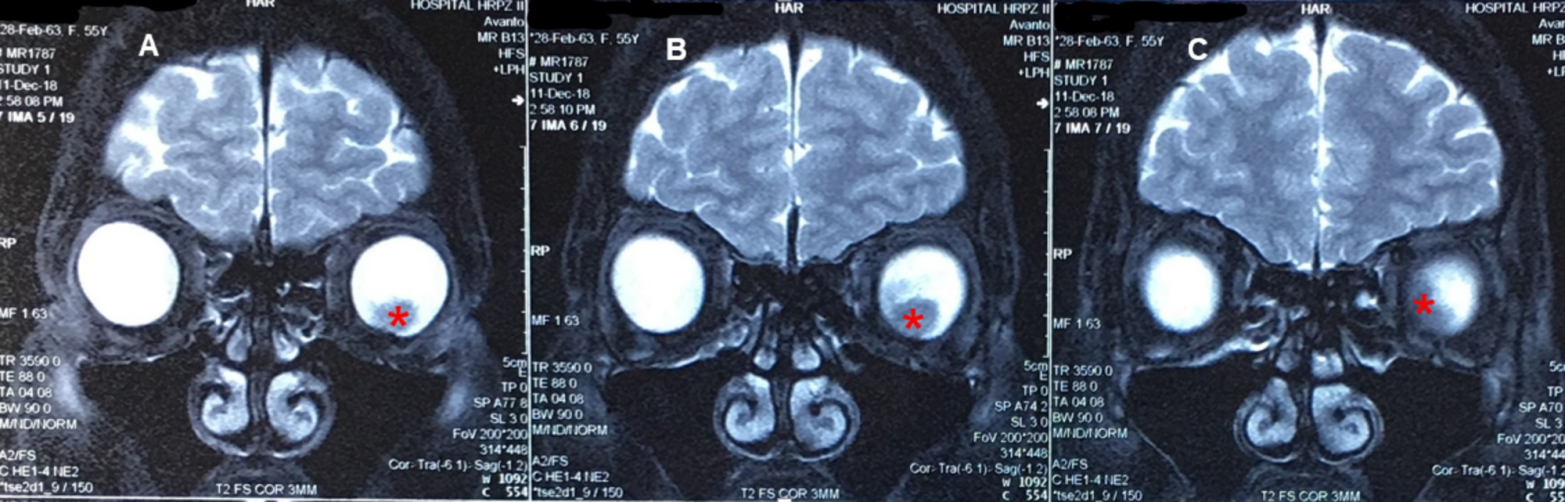
Coronal cut of MRI images taken at 3 mm intervals from anterior to posterior Image A (most anterior) showed the presence of a left intraocular mass inferiorly, appearing hypointense to vitreous in T2-weighted image (marked as *). In Image B, the lesion appeared larger in diameter and extended toward superonasal in a more posterior plane. Image C captured the mass at its posterior border. No extrascleral extension was noted in all planes.

B-scan demonstrated a hyperechoic choroidal mass at the posterior pole, with overlying huge subretinal fluid and retinal detachment (Figure 3). Magnetic resonance imaging (MRI) of the orbit and brain showed a subretinal lesion measuring 1.2 cm x 1.0 cm x 1.2 cm, associated with retinal detachment. The lesion showed an iso-hyperintense signal on T1-weighted image, hypo-intense signal on T2-weighted image, and enhanced post contrast. The lens and optic nerve were still preserved. No retro-orbital enhancing mass was seen. There was also an absence of an extrascleral extension or intracranial lesion (Figure 4). The test for tumor markers showed negative results (Table 1).

**Table 1:** Tumor markers were all within the normal range

Tumor Markers	Result
Cancer Antigen 125 (CA 125)	15.1 U/ml (Normal)
Carcinoembryonic Antigen (CEA)	1.1 ug/L (Normal)
Lactate Dehydrogenase (LDH)	198 U/L (Normal)
Alpha-Fetoprotein (AFP)	2.3 IU/mL (Normal)

Additional opinions from both oculoplastic and medical retina specialists were sought regarding diagnosis and treatment plans. The patient was then diagnosed with choroidal melanoma and planned for enucleation of her left eye. Unfortunately, the enucleation was not yet carried out, as she opted to delay the operation. She is otherwise fully aware of the possible consequences in the absence of timely intervention. Surveillance computed tomography (CT) of the thorax, abdomen, and pelvis was also done and showed no distant metastasis. Her liver enzymes were within normal range.

## Discussion

Choroidal melanoma usually presents with exudative retinal detachment. A rhegmatogenous retinal detachment is a very rare presentation, accounting for less than 1% of all choroidal melanoma cases. Hence, in the absence of a risk factor to get malignant melanoma, the presentation of an acute bullous retinal detachment is typically related to a retinal tear following degenerative myopic changes. A subretinal lesion is almost impossible to be visualized in the presence of a bullous retinal detachment, as what we observed in this patient; hence, this is the possible reason of a nearly missed diagnosis of choroidal melanoma.

A diagnostic challenge is not uncommon in this type of disease especially when it is frequently associated with masquerade syndrome [11]. At an earlier stage, the risk of a tumor being overlooked is also present. In a study by Damato, most of the subjects requiring primary enucleation had been misdiagnosed at initial review, leading to a long delay in treatment [12].

Our patient was asymptomatic until she developed extensive exudative retinal detachment, as she had been experiencing reduced vision with superior visual field loss. This was in concordance with what had been described by Damato, that one-third of patients diagnosed at an earlier stage of disease were asymptomatic, whereby the lesion was picked up incidentally. Meanwhile, the rest did experience symptoms such as blurred vision (38%), photopsia (9%), floaters (7%), visual field loss (6%), visible tumour (3%), pain (2%), and metamorphopsia (2%) [12].

When visualization of the mass is permissible, choroidal melanoma appears as a dome-shaped mass in a majority of cases. Other than that, it may assume a mushroom configuration following the rupture of Bruch’s membrane (19%) and, rarely, a lesion of choroidal melanoma presented as a diffuse variant (6%). The lesion can be pigmented, non-pigmented, or have a mixed color. Other characteristic features of choroidal melanoma include the presence of orange pigment (lipofuscin) at the level of retinal pigment epithelium [13].

At present, a diagnosis of choroidal melanoma was made based on clinical grounds supported by imaging, as an intraocular biopsy for diagnostic purposes is not possible without enucleating the affected eye. Tara has described the safety of intraoperative fine-needle aspiration biopsy during plaque surgery, but it was done with the aim to obtain prognostic information [14]. Ultrasonography of the tumor will show a high initial spike and low to medium internal reflectivity with decreasing amplitude, followed by a prominent spike corresponding to the sclera in A-scan while B-scan usually demonstrates a choroidal mass characterized by acoustic hollowness and choroidal excavation [11]. Magnetic resonance imaging (MRI) showed the lesion appeared to be hyperintense to vitreous on T1-weighted image and hypo-intense to vitreous on T2-weighted image.

Choroidal melanomas were frequently found to be associated with subretinal fluid, intraocular hemorrhage, or extraocular extension. Apparently, two-thirds of cases were associated with various degrees of exudative retinal detachment, especially in a large lesion of greater than 4 mm width [15-16]. Our patient presented with a huge mass of 12 mm, thus the complication of extensive exudative retinal detachment was almost inevitable. Subretinal fluid accumulation occurs due to disrupted fluid hemostasis across the retinal pigment epithelium, and it may range from localized to almost total retinal detachment. Symptomatic exudative retinal detachment indicates retinal layer separation of the fovea and has a poorer visual prognosis [8]. It is important to note that any presentation of retinal detachment demands a thorough evaluation, especially when the distribution of subretinal fluid appears to be unusual and does not follow the typical rule.

Apart from exudative retinal detachment, other, common, associated secondary changes include atrophy of the retinal pigment epithelium and neurosensory layer, cystoid retinal degeneration leading to retinoschisis, and invasion of the sensory retina by the tumor [17]. The presence of exudative retinal detachment has no direct correlation with the staging of the disease, but it is more likely to present in larger tumor size, may pose a substantial risk for metastasis, and be responsible for a majority of cases of visual loss [18]. It presents in more than half of the patients and is a risk factor for local treatment failure [19]. Muscat et al. reported that all of their 20 studied untreated choroidal melanoma subjects were detected to have subretinal fluid using time-domain optical coherence tomography [17]. As nowadays, we embark on the globe conserving treatment more often than enucleation, timely vitreoretinal surgical intervention to treat exudative retinal detachment has been suggested as a measure to reverse visual loss. Gibran proved that successful treatment of exudative retinal detachment was able to improve vision, which appeared to be contradictory to previous reports of irreversible visual loss after exudative retinal detachments secondary to photoreceptor atrophy [7].

In terms of prognosis, survival rate correlates with primary tumor size. However, approximately 50% of patients will develop metastatic disease [2]. Current local treatment or a globe-preserving procedure does not offer extra benefits in terms of metastasis prevention and longevity of life as compared to enucleation [4].

## Conclusions

Early diagnosis of choroidal melanoma is vital so that appropriate treatment can be initiated without delay. However, many lesions are known to simulate this tumor, making timely diagnosis greatly challenging.
